# Ultrasonographic assessment of renal microcirculation is a new vision for the treatment of intensive care unit associated acute kidney injury

**DOI:** 10.1186/s40001-024-01704-y

**Published:** 2024-02-10

**Authors:** Rongping Chen, Beijun Gao, Xinchen Wang, Hua Zhao, Xiaoting Wang, Dawei Liu

**Affiliations:** Department of Critical Care Medicine, Peking Union Medical College Hospital, Peking Union Medical College & Chinese Academy of Medical Sciences, Beijing, China

**Keywords:** AKI, Microcirculation, CEUS, Kidney regional blood flow

## Key points

- Microcirculatory impairment is a key pathogenetic mechanism in acute kidney injury associated with severe disease.
- CEUS is a "microscope" of the renal microcirculation.
- CEUS quantitatively assesses the renal microcirculation to help guide hemodynamic therapy in critical care-related acute kidney injury.
- CEUS is challenging but necessary to differentiate regional blood flow within the kidney.
- CEUS parameters can be used as sensitive indicators to assess the prognosis of AKI.

## Introduction

Acute kidney injury (AKI) refers to a clinical syndrome that occurs as a result of a rapid decline in renal function caused by a variety of etiological factors. AKI is a common complication in critically ill patients, increasing length of hospital stay, hospitalization costs, and morbidity and mortality. In the Intensive Care Unit (ICU), sepsis, cardiac critical illness, hypovolemia, abdominal hypertension, and urinary tract obstruction are among the critical care-related factors that can cause AKI. The pathophysiological mechanisms of AKI are not exactly the same for different critical care etiologies. Altered perfusion is considered an important pathogenesis of AKI, and whether optimization of microcirculation is achieved after optimization of microcirculatory hemodynamics is a blind spot for clinicians. In recent years, contrast-enhanced ultrasound (CEUS) is an emerging imaging technique in the field of critical illness using highly echogenic but inert microbubbles to delineate areas of microvessel perfusion within organs, which has been widely used in oncology and other fields, and for ICU patients, CEUS can quantitatively assess the alterations in renal microcirculatory blood flow. Ultrasound microbubble contrast agent can be injected via peripheral vein through the pulmonary circulation to finally reach the target organ or tissue, to achieve tissue echo enhancement, and the contrast agent can be observed in the tissue of the area of interest. Evaluation of renal microcirculation using ultrasonography allows early detection of people at high risk of AKI, early intervention to avoid the occurrence and development of AKI, and organized and individualized hemodynamic therapy. Therefore, this review discusses the epidemiology of critical illness-related AKI, pathophysiological mechanisms, ultrasonography agents and their techniques, renal ultrasonography procedures and their applications, including the operational steps, renal ultrasonography images at different periods, and the meaning of CEUS parameters. Finally, the current applications of ultrasonography in severe disease-related AKI, especially the evaluation of intrarenal regional blood flow, are summarized.

### Epidemiology of acute kidney injury in ICU

Acute kidney injury (AKI), with a prevalence of up to 20–50% and a morbidity and mortality rate of > 10%, increasing the length of hospital stay, hospitalization costs, and morbidity and mortality rates, refers to a clinical syndrome that occurs as a result of a rapid decline in renal function caused by a variety of etiological factors [1–3]. There is growing evidence that the burden of AKI extends beyond progression to chronic kidney disease, increased risk of cardiovascular complications, recurrent episodes of AKI, and increased long-term mortality [4]. Due to the complex etiology and specific pathogenesis that remain unclear, there are limited means of clinical assessment and a lack of specific treatments. Treatment to prevent the development or progression of AKI is currently limited to optimizing hemodynamic and fluid status, avoiding nephrotoxins, and the search for a specific pharmacological treatment is hampered by diagnostic delays and complex, incompletely elucidated pathophysiology [5]. Defining the pathophysiological mechanisms of AKI, closely monitoring renal hemodynamics and optimizing the hemodynamic state are essential for the diagnosis and treatment of AKI.

### Microcirculation as a key pathogenic mechanism in ICU associated AKI

**Fig. 1:**
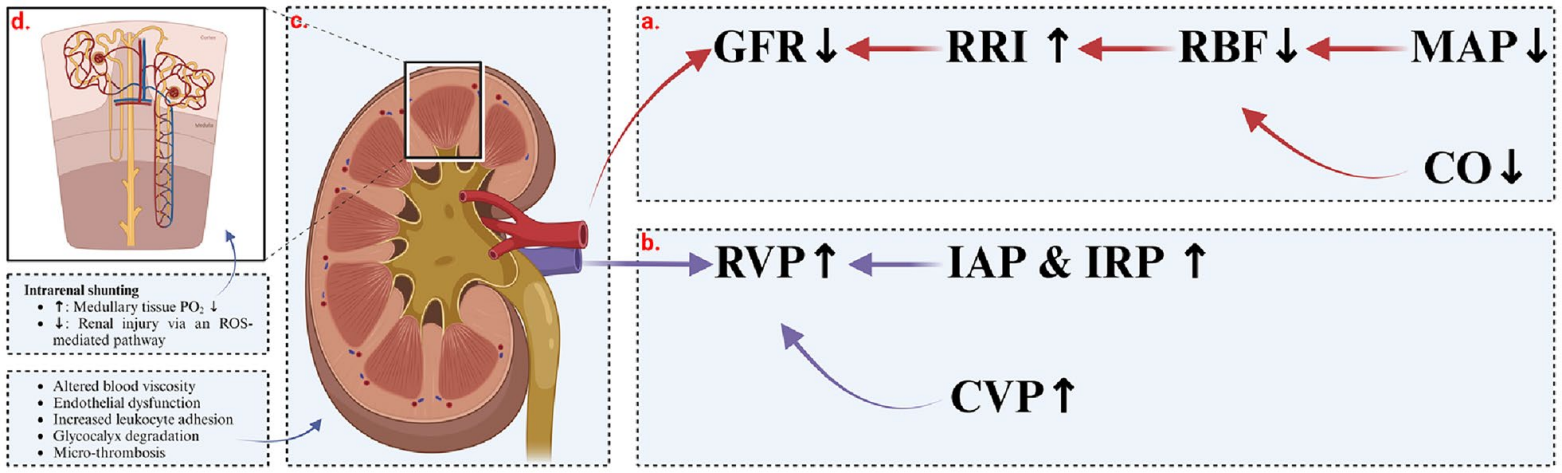
Diagram of renal hemodynamic mechanisms. **a** Reduction in mean arterial pressure (MAP) or cardiac output (CO), which determines renal preload, leads to decreased renal blood flow (RBF). This triggers an increase in renal arteriolar resistance (RRI), abnormally high in this context, resulting in a lowered glomerular filtration rate (GFR). This change adversely affects renal microcirculatory blood flow, contributing to AKI. **b** Increased central venous pressure (CVP), intra-abdominal pressures (IAP), or renal interstitial pressures (IRP) elevate renal venous pressure (RVP). **c** Discrepancies between renal macrocirculation and microcirculation may arise from various factors, including altered blood viscosity, endothelial dysfunction, increased leukocyte adhesion, glycocalyx degradation, and micro-thrombosis. **d** Enhanced intrarenal shunting can lead to reduced medullary tissue PO_2_, causing varying levels of tubular hypoxia. Conversely, a reduction in shunting effectiveness may also induce renal injury via a reactive oxygen species (ROS)-mediated pathway

In the ICU, AKI arises from diverse critical etiologies, each with unique pathophysiologic mechanisms (Fig. 1). Microcirculatory dysfunction, inflammatory dysregulation, and metabolic reorganization are the main causes of renal tubular dysfunction [6]. Alterations in renal perfusion underlie the development of AKI. The kidney has an exceptionally high perfusion rate to maintain a high glomerular filtration rate (GFR). Blood flow enters the kidney through the cortex, with 90% supplied to the cortex, while the medullary blood volume perfuses cortical tissue first, so its proportion is part of the cortical blood volume. The presence of intra-renal shunts makes the medulla more susceptible to hypoxia, and medullary hypoxia resulting from redistribution of intra-renal flow plays an important role in the development of AKI [7–9]. Many studies are proving that renal blood flow is increased in the early stage of sepsis, and GFR is decreased due to the abnormal distribution of intrarenal blood flow and the different degree of dilatation of glomerular inlet and outlet arterioles. Whereas sepsis is a dysregulated immune-inflammatory response leading to damage to the vascular endothelium, inappropriate positive fluid balance is central to the further development of sepsis-associated AKI, with dysfunction due to alterations in vascular endothelial cell permeability being the most important pathophysiological mechanism [10, 11]. The kidney serves as an important window for organ perfusion monitoring in ICU patients [12]. Sepsis associated AKI is actually a microcirculatory disease in which inadequate perfusion and hypoxia of renal tissues are emerging as key mediators in the pathogenesis of AKI [13]. Intrarenal hypoxia may result from severely reduced renal blood flow (RBF) and altered intrarenal hemodynamics, for example, renal tubular swelling leading to renal microvascular compression [9]. Using a porcine sepsis induction model, Lima and colleagues demonstrated that renal and subglottic microcirculatory injury persisted after normalization of cardiac output (CO) [14]. Recent studies on post-cardiac surgery patients also link the development of AKI with inadequate renal medullary perfusion and microcirculatory disorders [15]. In severe sepsis, porcine models showed reduced renal cortical microcirculatory blood flow preceding changes in RBF [16]. A rat model similarly demonstrated that during sepsis and septic shock, microcirculatory alterations in peripheral mucosa and kidneys precede overall hemodynamic changes [17].

The goal of clinical hemodynamic resuscitation is to meet the oxygen and metabolic demands of each organ, which can only be achieved by optimizing the microcirculation [18]. However, studies in patients with septic shock have found that microcirculatory damage is more severe and long-lasting in the kidneys [19]. This shows that there is a difference between macro-circulatory and microcirculatory blood flow, even in different regions [20–22]. However, optimization of the macro-circulation may not improve tissue perfusion in the presence of alterations within the microcirculation, including altered blood viscosity, endothelial dysfunction, increased leukocyte adhesion, glycocalyx degradation, and micro-thrombosis [23]. Another risk is the over-optimization of the macro-circulation leads to fluid overload or overuse of vasopressor drugs, which is often deleterious in terms of tissue oxygenation [24]. Clinicians currently pay insufficient attention to organ microcirculation, which prevents them from individualizing resuscitation by targeting the microcirculation.

Abnormal intrarenal shunting is also one of the important hemodynamic mechanisms of acute kidney injury. The kidney’s unique vascular anatomical arrangement facilitates arterio-venous oxygen shunting. This arrangement possibly serves to maintain a stable renal tissue partial pressure of oxygen (PO_2_) in the presence of variable RBF [25]. However, it can also cause low medullary tissue PO_2_ when shunting is enhanced, leading to a variable degree of tubular hypoxia. Alternatively, a reduction in shunting effectiveness may also cause renal injury via reactive oxygen species (ROS)-mediated pathway [26, 27]. In a sheep model of septic hyperemic renal dysfunction that reduced medullary blood flow and oxygen tension preceded the decrease in urine output and creatinine clearance [8]. Watchorn et al. demonstrated that the severity of AKI was independent of the degree of renal cortical under-perfusion in patients with septic shock [19]. Treatment with norepinephrine normalized mean arterial pressure (MAP) and increased RBF but further reduced medullary perfusion and oxygenation, and that medullary perfusion appeared to be independent of RBF and cortical perfusion [28]. Renal medullary hypoxia due to redistribution of intrarenal perfusion is thought to be a key mediator of sepsis-related AKI [29].

These findings indicate a mismatch between macrocirculation and microcirculation, the existence of intrarenal shunts, and distinct responses of renal cortical and medullary blood flow under varying conditions. Thus, close monitoring of alterations in renal cortical and medullary microcirculation and a focus on hemodynamic therapy targeting renal microcirculation are vital for diagnosing and treating severe AKI associated with serious illnesses.

### The key techniques in assessment of the renal microcirculation

Various methods exist for monitoring renal microcirculation, including invasive laser Doppler, non-invasive imaging techniques, and other imaging modalities that might negatively impact renal function. These approaches, however, are limited by their invasiveness, limited bedside availability, and potential adverse effects on renal function [14, 30–33]. Previous studies have shown that partial pressure of urine oxygen (PuO_2_) is closely correlated with medullary oxygen concentration and has been described as a clinical window into renal medullary health [34, 35]. Notably, reduced PuO_2_ following extracorporeal circulation in cardiac surgery could be associated with the subsequent development of AKI. Improving renal medullary oxygenation during such procedures might attenuate postoperative AKI [36]. However, factors like stagnant urine and low flow rates can influence PuO_2_ measurements, compromising their ability to accurately reflect renal medullary oxygenation [35, 37]. Therefore, simple and accurate means of monitoring renal microcirculation are needed to guide renal hemodynamic therapy.

Doppler ultrasound, widely recognized as a critical tool in the ICU, is the most common method for clinical evaluation of renal blood flow [38]. CEUS is a technology that can observe organ vascular perfusion in real time, and it can show renal perfusion at different times and in different parts of the kidney [39]. CEUS specifically targets the renal cortex and medulla within the region of interest (ROI), extracting parameters of renal microcirculatory perfusion through a perfusion curve model derived from intensity-over-time data. Its application in studying renal microvascular perfusion is well-documented in both animal models and human subjects. The parameters obtained through CEUS demonstrate strong correlation well with the gold standard measures of renal blood flow, such as para-aminohippurate clearance, in studies involving healthy volunteers [14, 40–45]. This method is particularly useful for bedside monitoring of organ microcirculation in patients, providing quantitative assessments of renal perfusion [24, 46–48]. CEUS employs ultrasonographic contrast agents consisting of small gas-filled encapsulated microbubbles. Since these microbubbles are confined to the vascular system, CEUS offers a unique approach for visualizing microcirculation and quantifying blood flow. This technique has notable advantages for real time, bedside imaging [49–51]. In addition, CEUS’s reproducible characteristics facilitate individualized monitoring of renal microcirculatory perfusion as needed [52]. In various animal studies, CEUS has shown a consistent pattern of renal perfusion enhancement, initially in the renal cortex and subsequently in the medulla. This pattern enables differentiation between cortical and medullary blood flow in kidneys [53–56]. The safety of CEUS has been established through several large retrospective studies, including those involving critically ill patients [57–59].

Microbubble ultrasound contrast agents (UCAs) consist of microbubbles in suspension that interact strongly with the ultrasound beam and are easily detected by ultrasound imaging systems [60]. Currently, great progress has been made in the development of UCAs, mainly in the reduction of the diameter of UCAs (< 8 μm), which allows them to be injected via a peripheral vein through the pulmonary circulation to ultimately reach the target organ or target tissue and achieve tissue echo enhancement, and in the increasing stability of ultrasound microbubble contrast agents, which allows the contrast agent to be observed in the tissue of the region of interest [61]. Presently, the FDA has approved three UCAs for intravascular use: Optison, Definity, and SonoVue [60]. SonoVue is predominantly utilized in Europe [62–64]. Excellent contrast agents have the following characteristics: high safety and low side effects; uniform microbubble size with a diameter of less than 10 μm and can be controlled; free passage through capillaries with hemodynamic characteristics similar to those of red blood cells; the ability to generate rich harmonics; and good stability [60]. UCAs are particularly beneficial for patients with compromised renal function as they are non-nephrotoxic and do not induce renal tissue damage [65, 66].

Bedside CEUS enables hemodynamic organization assessment for the treatment of critically ill patients, monitoring renal microcirculatory blood flow dynamically and continuously, and guiding hemodynamic therapy, thus enabling individualized and organized treatment.

### Standardized practice and parametric significance of renal CEUS—core prerequisites

Assessment of renal microcirculatory perfusion consists of several parameters [67]. Qontraxt and Sonotumor software analyses the main parameters of time–intensity curve (TIC) of ultrasonography are slightly different (Table 1) [48, 68]. The CEUS parameters correlate with the vascularization of the area analyzed. The time-based variables are more representative of blood flow, whereas the intensity-based variables are suggested to represent blood volume within a specific region. All time and intensity values were calculated from fitted curves rather than from raw image data [50, 69]. For reproducibility studies of CEUS parameters, it was found that in healthy cat kidneys, cortical time parameters had a low coefficient of variation and were reasonably reproducible, whereas intensity parameters and medullary-related parameters were poorly reproducible [70]. In healthy dogs it was also found that time parameters of CEUS had the least variability [71]. Good reproducibility of the parameter mean transit time (MTT) in CEUS assessment of renal cortical perfusion was found in healthy adults [52]. Renal cortical MTT also showed good agreement among different observers [72]. Averkiou et al. compared different software for analyzing contrast TIC and found that rise time (RT) and MTT were reproducible, whereas peak intensity (PI) and area under the curve (AUC) were more variable [73]. There are many factors affecting the results of quantitative analysis of ultrasonography, and the quantitative parameters obtained at different depths are different, and some studies have shown that only the quantitative parameters at the same depth are comparable; meanwhile, the stability of the quantitative parameters obtained at a depth of 4–6 cm is the best; while the size and shape of the ROI have no effect on the quantitative parameters. Different selected sites of the lesion will also affect the results of the analysis of the contrast parameters, thus not accurately reflecting the blood supply of the lesion. Differences in contrast dose and injection rate can significantly affect time to peak (TTP), PI, etc., and therefore factors that may have an impact should be avoided during ultrasonography [74]. The interindividual heterogeneity of CEUS measurements should be attributed to the conditions of acquisition in ICU: renal cortical depth based on the patient's body mass index, respiratory movements that may change the plane of the acquired ultrasound, and changes in tissue thickness and echogenicity over time due to an increase in body fluid balance. of tissue thickness and echogenicity. Such heterogeneity has been reported in previous studies of ICU patients; however, despite this heterogeneity, changes in renal cortical perfusion are readily detectable [47, 75].

**Table 1:** Main component parameters of TIC

Software	Parameters	Parameter abbreviation	Parameter name	Unit	Definition	Significance	References
Qontraxt software to analyze ultrasonography time–intensity curve parameters	Time parameters	AT	Arrival time	s	Time after injection when ROI signal start to enhance	Determined by the blood flow velocity in renal cortical microvessels	Ma et al. [68]; Luo et al. [69]; Seo et al. [77]
		TTP	Time to peak	s	Time after injection when ROI signal intensity reaches its maximum	It is the time from zero intensity to maximum intensity. This parameter is calculated from the fitted mathematical model and often is supplied in a closed form analytical expression	
		AS	Ascending slope	dB/s	Slope of ascending part of TIC	Representing the perfusion speed of the ROI	
		DT/2	Descending time/2	s	Half of descending time	Time needed after injection for intensity to decrease to half of PI	
		DS	Descending slope	dB/s	Slope of descending part of the TIC, representing the dilution speed of the ROI	Reflecting the total number of microbubbles clearing the vessels within the ROI, in response to renal perfusion	
	Intensity parameters	PI	Peak intensity	dB	The peak intensity is the difference between the maximum and minimum intensity	Reflecting the total number of microbubbles entering the vessels within the ROI, in response to renal perfusion	
		AUC	Area under the curve	dB/s	Area under the TIC curve	Influenced by blood flow velocity and blood distribution volume, it is proportional to the mean blood flow at the ROI, which reflects changes in intravascular blood flow volume	
Sonotumor software analyses contrast time–intensity curve parameters	Time parameters	RT	Rise time	s	The time from injection until the peak of enhancement	Referring to the time interval between the first arrival of contrast and TTP	Schneider et at. [76]; Harrois et al. [10]; Liu et al. [78]; Nylund et al. [79]
		MTT	Mean transit time	s	Describe the average time it takes for a microbubble to pass through the ROI	It is a measure of the time to recharge after contrast destruction, with shorter times indicating higher levels of perfusion	
		FT	Fall time	s	Referring to the duration of contrast wash-out	–	
		WIS	Wash in slope	dB/s	The speed from the beginning of enhancement to the peak of enhancement	Maximum of wash in slope of contrast agent	
		WIT	Wash-in time	dB/s	It is time from 5% intensity to 95% intensity	It is proportional to the time to peak but it is sometimes used with mathematical models that do not have closed form analytical expressions of TTP	
		WOT	Wash-out time	dB/s	It is the time from the peak of the TIC curve to the zero value again	The latter timepoint (zero enhancement) is rarely seen in the raw data as it may take a long time for the ROI to become completely black again. It is easily calculated from the fitted mathematical model (curve)	
		WiPI	Wash-in perfusion index	dB/s	Calculated as WiAUC divided by RT	–	
	Intensity parameters	RBV	Relative blood volume	a.u	Measure of maximum intensity of ROI after full recharge	RBV is proportional to the concentration of contrast agent within the ROI and increases with increasing perfusion levels	
		PI	Perfusion index	a.u	RBV/MTT	A measure of maximum CEUS signal intensity that is more variable than time to replenishment	
		PE	Peak enhancement	a.u	The maximum intensity of the TIC		
		WiWoAUC	Wash-in and wash-out area under the curve	dB/s	The total area under the curve of TIC	-	
		WiAUC	Wash-in area under the curve	dB/s	The area under the TIC from time of arrival to the PE	-	
		WoAUC	Wash-out area under the curve	dB/s	The area under the TIC from the PE to the end of the curve	–	

*TIC* time–intensity curve, *ROI* region of interest, *a.u.* arbitrary units

**Fig. 2:**
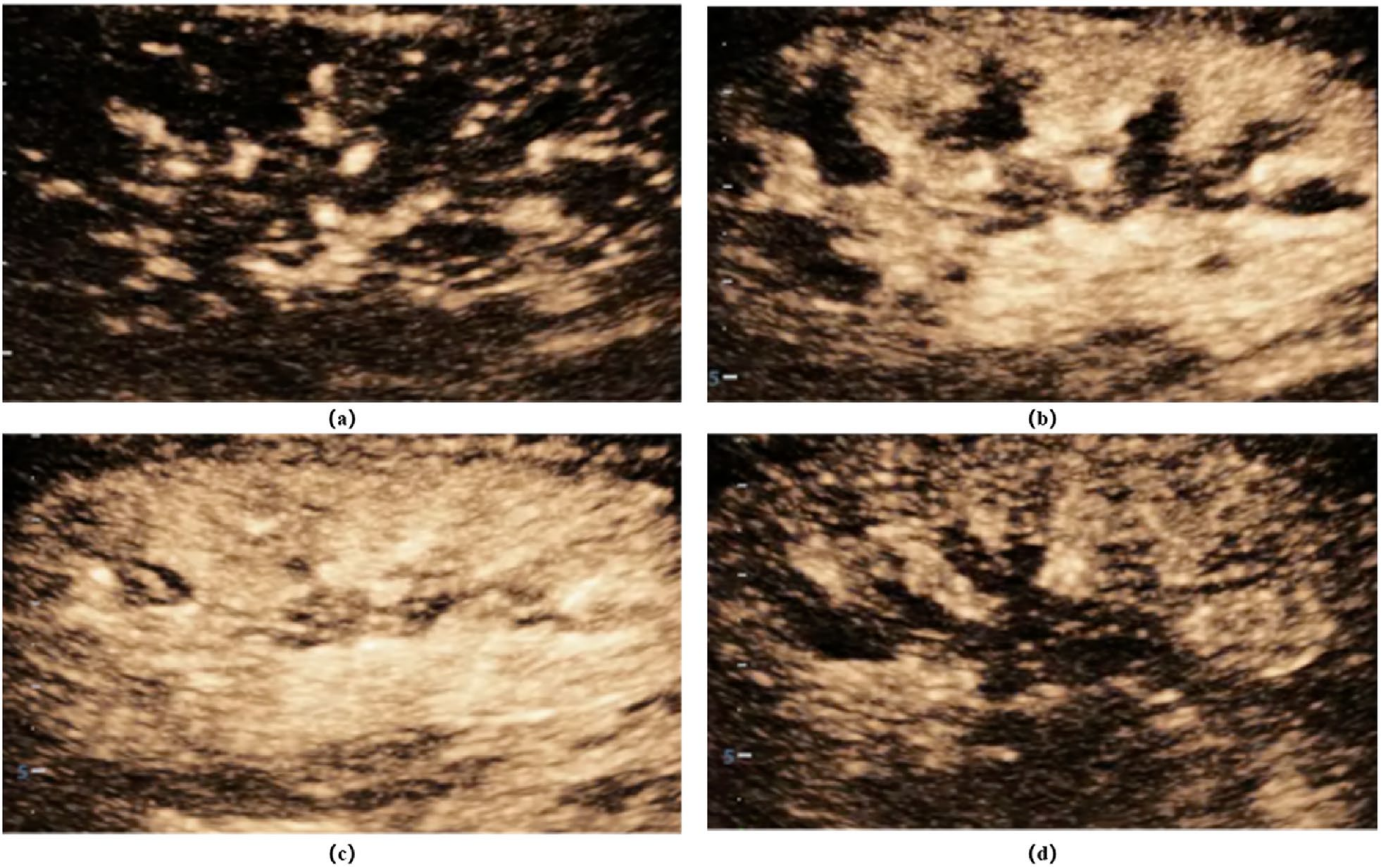
**a** 5–10 s of injected contrast agent microbubbles of contrast agent reached the interlobular arteries and arcuate arteries; **b** 10–15 s the renal cortex began to develop and gradually enhanced, and the renal medulla did not develop; **c** renal medulla was developed and gradually enhanced after 25 s; **d** it began to gradually fade after about 1 min and lasted for about 3–6 min. The above images were derived from our own patients which was approved and agreed the requirement for informed consent by the institutional review board of Peking Union Medical College Hospital (approval number, I-23PJ1284)

**Fig. 3:**
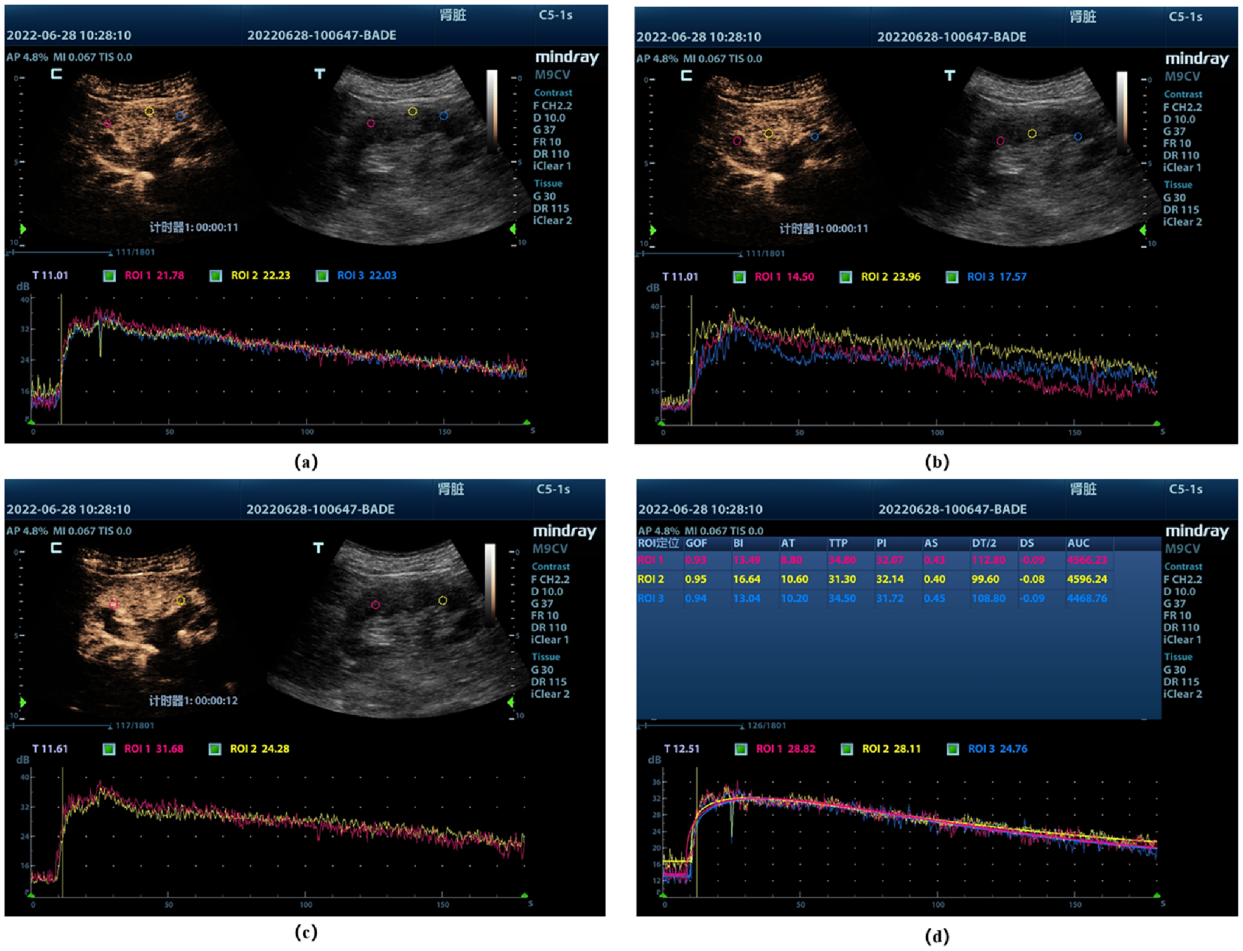
Time–intensity profiles of different sites. **a** Cortical sampling site and TIC, the screen shows the contrast-enhanced image (C) As well as the conventional ultrasound image (T). **b** Medullary sampling site and TIC. **c** Corticomedullary junction sampling site and TIC. **d** Plotting of regions of interest (ROIs) analyzed by the software, and then generating the supply curves for each ROI (lower section), these curves represent intensity as a function of time. The above images were derived from our own patients which was approved and agreed the requirement for informed consent by the institutional review board of Peking Union Medical College Hospital (approval number, I-23PJ1284)

Standardized ultrasonography procedures and skilled ultrasound techniques are prerequisites for us to obtain accurate data. CEUS uses a dual-image display format with a low mechanical index (MI < 0.1). This format allows two views to be displayed in parallel; the contrast view is constructed by selectively filtering the signal to identify microbubbles while excluding background signals, so that the initial image is blank before contrast is injected. A standard grey-scale image displayed simultaneously identifies the kidney prior to infusion. Low MI ultrasound is necessary to prevent microbubble destruction [48]. Contrast agent use is divided into two methods: infusion and push. In the continuous infusion method, 4.8 ml of SonoVue contrast agent is infused at a rate of 1 ml/min with a special infusion pump until the total amount is infused [76]. The intravenous push method involves a one-time infusion of a certain amount of contrast agent through a central vein (Table 2). It has been found that parameters related to quantitative perfusion by the intravenous push method are reproducible across instruments and analysis software [73]. Different renal ultrasonography images were shown at different times due to the different development times of CEUS in the renal cortex and medulla (Fig. 2). Cortical, medullary and even corticomedullary junction ROIs were sampled (Fig. 3), and since the maximum number of sampling points was 8, 3 ROIs were sampled for cortex and medulla, and 2 ROIs were sampled for corticomedullary junction, with one point at each of the upper, middle and lower poles of the kidneys, respectively, to ensure that they were taken at the same time and to better discriminate between the different parts of the sample.

**Table 2:** Specific procedures for renal ultrasonography

Step	Content
1	Establishment of intravenous access
2	Select the appropriate probe, abdominal probe is recommended, obtain a standard long-axis view of the kidney, adjust the image depth, focus and frame rate
3	Entering contrast mode
4	The contrast agent was added to 5 ml saline according to the instructions and shaken well. The median elbow vein was injected with 0.02 ml/kg of contrast medium and 10 ml of saline washed through the tube (there are recommendations of 1.0–2.0 ml, which vary from unit to unit)
5	Simultaneous timing and image recording
6	Sampling sites (regions of interest): three each from the cortex, medulla and corticomedullary junctional zone (avoiding large vessels)
7	Image Acquisition and Analysis
8	Completion of contralateral nephrography in 20 min

### Application of CEUS in ICU-related AKI—with emphasis on intrarenal regional blood flow

#### Normal CEUS renal manifestations

There has been more studies on the application of CEUS (Table 3). As previously discussed, corticomedullary blood flow distribution is uneven, with the medulla receiving less blood supply and being more susceptible to hypoxia [8]. Comprehensive monitoring of intrarenal regional blood flow in different disease states is needed to individually titrate blood pressure targets and find the optimal perfusion pressure to ensure renal perfusion. CEUS has become a crucial technique for bedside assessment of intrarenal regional blood flow in the ICU. This method enables the differentiation of blood flow in the intrarenal cortical, medullary, and corticomedullary junctional zones. This differentiation is possible given the difference in the timing of contrast agent arrival at the cortex and medulla [80]. However, research on the application of CEUS for assessing renal microcirculatory blood flow in ICU patients with AKI exhibits considerable variability. Some studies indicate that CEUS is capable of measuring cortical perfusion, but the assessment of renal medullary blood flow is limited by technical challenges [19]. It has also been shown that there are differences in CEUS measurements of renal cortical and medullary blood flow, and that the changes and significance of blood flow parameters differ between regions. In healthy cats, PI, wash in slope (WIS) and MTT parameters were significantly higher in the renal cortex than in the medulla [54]. In ICU patients, renal cortical TTP parameter and medullary rise time (RT) parameter may contribute to the diagnosis of AKI [81].

**Table 3:** Apply of CEUS in AKI

Category	Refences	Model	Intervention	CEUS parameters	Key findings
Intrarenal regional blood flow applications	Brabrand et al. [83]	Pigs	Hypoxia	Cortical PI↓, medullary TTP and MTT↑	Global hypoxia induced changes in overall and regional renal perfusion detectable with CEUS
	Komuro et al. [92]	Rats	Bolus injection of normal saline	Cortical and medullary TTP↑, and cortical PI ↓in the CVP 15 mmHg group; medullary PI ↓in CVP 10 mmHg group	Impaired renal parenchymal flow accompanied with increased renal interstitial pressure
	Komuro et al. [91]	Patients with congestive heart failure	Decongestive therapy	cortical TTP ↓, medullary TTP (–)	Renal congestion can be observed using CEUS
	Song et al. [81]	ICU patients	–	Cortical TTP↑, medullary RT↑	Can aid the diagnosis of AKI in ICU patients
	Li et al. [104]	AKI patients	–	WIR↓, MTT and PT ↑	Reduced microcirculatory perfusion had occurred in AKI patients prior to the alteration of blood creatinine
	Wang et al. [105]	AKI patients	–	PIT and WIR↓, PT↑	Compared to SCr and BUN, CEUS parameters can early response to renal dysfunction
	Yoon et al. [102]	AKI and non-AKI patients	–	Cortical RT, MTT, RT and WIS; medullary RT and PI; AUC of cortical and medullary	Diagnosing the severity of AKI and predicting renal prognosis
	Liu et al. [103]	Septic patients	–	PT, AS, DT/2 and MTT↑	Assessment the possibility of severe AKI
	Liu et al. [106]	Septic AKI patients	–	PI↓, TTP↑	CEUS is of great help in the detection of condition changes and prognosis
	Wang et al. [107]	Septic AKI patients	–	RT↑, PI and WIS↓	Combination of blood creatinine, WIS and PI improved the accuracy of diagnosing AKI
	Harrois et al. Watchorn et al. [10, 19]	Septic shock patients	–	Cortical PI and WIR↓; MTT ↑	Renal cortical hypoperfusion is a persistent feature in critically ill septic patients who develop AKI
Application of CEUS in AKI prognosis and its influencing factors	Schweiger et al. [54]	Healthy cats	–	PI, WIS, MTT	Higher in the renal cortex than in the medulla
	Schneider et al., [75, 101]	Colorectal surgery and cardiac surgery patients	–	PI↓, MTT↓	Predict postoperative renal adverse events
	Luo et al. [69]	I/R rabbits	–	AT and TTP values peaked 3 d	Correlated with the most significant pathological changes at the same timepoint
	Schneider et al. [110]	Healthy subjects	Ang II	Cortical PI↓	
	Imamura et al. [111]	Healthy subjects	Diclofenac sodium	Cortical PI↓	
	Dong et al. [117]	Healthy rabbits	Nitroglycerin	Cortex TTP and AUC↑; AS and DS↓	–
	Haers et al. [118]	Dogs	Hydrocortisone	Cortical and medullary PI↑	–
	Wang et al. [112]	CLP rats	Curcumin	PI, AUC, and DT/2↑	Improve renal microcirculation
	Si et al. [114]	I/R rabbits	Dexmedetomidine	PI↑, TTP and AUC↓	Improve renal microcirculation
	Stock et al. [115]	I/R cats	Ang II	WiPI, WOR, and WiAUC↓	–
	Ergin et al. [116]	Severe hemodilution pigs	Hydroxyl ethyl starch	Cortex MTT↓	Preserved intrarenal microcirculatory perfusion and renal function
	Wang et al. [109]	Septic shock patients	Terlipressin	PI↑	Improve renal perfusion

“↓” decline, “↑” increase, “–” unchanged

*CEUS* contrast-enhanced ultrasound, *PI* perfusion index, *MTT* mean transit time, *WIS* wash in slope, *TTP* time to peak, *CVP* center vein pressure, *ICU* intensive care unit, *CLP* cecum ligation and puncture, *AKI* acute kidney injury, *RT* rise time, *RRT* renal replacement therapy, *AUC* area under the curve, *PIT* peak intensity time, *AS* ascending slope, *DT/2* descending time/2, *WIR* wash-in rate, *AT* arrival time, *WiPI* wash-in perfusion index, *WOR* wash-out rate, *WiAUC* wash-in area under the curve, *DS* descending slope, *Ang II* Angiotensin II

#### Renal CEUS manifestations in arterial telangiectasia

The renal circulation has two sets of capillary networks, the first being the glomerular capillary network located in the renal cortex, which filters the entire blood volume and forms the second capillary network. The glomerular capillary network is flanked at both ends by small arteries, which result in much higher forward perfusion pressures in the glomerular capillary network than in most other organs, and the kidneys are more susceptible to fluctuations in MAP [82]. In a porcine lipopolysaccharide model showing prolonged alterations in renal microvasculature during shock, CEUS effectively measures dynamic changes in renal microvascular perfusion during shock and resuscitation, insufficient renal microcirculatory perfusion can be quantified by a decrease in peak enhancement, and a decrease in intra-renal blood flow can be measured by measuring microbubble transport between the mesangial arterioles of the renal cortex and the capillaries time is estimated [14]. CEUS detects overall and regional renal perfusion changes induced by systemic hypoxia. Cortical and medullary flow are differentially affected by hypoxia, as evidenced by a significant increase in medullary TTP and MTT parameters and a significant decrease in cortical PI parameter [83].

#### Renal CEUS manifestations in venous terminal anomalies

The second group of the renal capillary network is the peritubular capillaries located mainly in the medulla, and to reabsorb most of the glomerular filtrate, the pressure within the peritubular capillaries must be sufficiently low. Mildly elevated renal venous pressure leads to increased renin release, resulting in increased renal vascular resistance and decreased renal perfusion [84]. In this case, higher positive perfusion pressure is required to increase renal perfusion, which makes the kidneys more susceptible to fluctuations in MAP and contributes to the onset and progression of AKI [85, 86]. Elevated CVP is the most direct factor affecting RVP and is an independent risk factor for the onset and progression of AKI [87, 88]. For the glomerular microcirculation, there is a curvilinear relationship between CVP and GFR, with GFR first increasing slightly and then decreasing sharply as CVP increases [88]. This subtle increase in GFR may be a reflection of increased cardiac filling to preserve cardiac function by Frank–Starling mechanism (pre-load), and subsequent renal perfusion [89]. While greater CVP levels will then decrease renal perfusion pressure, which will further impair GFR, because an increase in renal venous pressure can cause sodium retention by a direct action on the kidney: a rise in venous pressure could thereby initiate a vicious circle by causing sodium retention, expansion of plasma volume, and further increase in venous pressure [90]. Therefore, different pressures at the refluxing end of the kidney may have different effects on renal cortical and medullary blood flow. It has been shown that in patients with congestive heart failure, baseline TTP is significantly prolonged, and after decongestive therapy, renal cortical TTP decreases but medullary TTP remains unchanged [91]. In a rat model of renal stasis, it was found that renal cortical PI was significantly lower in the CVP 15 mmHg group of rats than in the control group. Whereas, renal medullary PI decreased in rats in CVP 10 mmHg group, but there was no statistical difference [92]. A recent study found that in patients with sepsis, CVP did not correlate with renal venous reflux flatus assessed by Doppler ultrasound, but the severity of renal venous stasis correlated with renal function [93]. In patients with heart failure, baseline renal venous return spectra can be used to predict adverse cardiorenal events [94]. Similarly, Beaubien-Souligny et al. found that venous stasis score, a five-prototypes of venous excess ultrasound (VExUS) grading system, which combines the diameter of the inferior vena cava and the venous Doppler waveforms of the portal, hepatic, and interlobular renal veins, was superior to CVP in identifying venous stasis in patients with post-cardiac AKI [95, 96]. Therefore, identification of renal venous stasis in ICU patients by ultrasound assessment of venous return may be superior to CVP.

### CEUS in the prognosis of renal function

CEUS has also been used to assess AKI prognosis, as a new vision for ICU microcirculation, a simplified method for assessing renal perfusion at the bedside, and correlates well with the gold standard renal blood flow measurements [42, 44, 97–100]. Schneider and colleagues first reported CEUS as a state-of-the-art technique for quantifying tissue perfusion and microcirculation capable of assessing renal cortical perfusion in ICU patients before and after cardiac surgery [75]. Furthermore, significant heterogeneity in renal cortical blood flow exists even in patients with similar degrees of AKI, suggesting that therapeutic interventions should ideally be based on an individual patient basis [19]. The value of CEUS in predicting postoperative renal adverse events has also been demonstrated in patients undergoing colorectal surgery and cardiac surgery [75, 101]. CEUS can be used as a tool for diagnosing the severity of AKI and predicting the renal prognosis of patients with AKI, in which cortical RT can predict AKI stage 3, MTT and RT can predict the initiation of renal replacement therapy (RRT); cortical WIS and medullary RT can predict the recovery of AKI; medullary PI and AUC predicted chronic kidney dysfunction progression; and AUC predicted RRT initiation and AKI recovery [102]. In patients with septic AKI, especially severe AKI, parameters peak time (PT), ascending slope (AS), descending time/2 (DT/2) and MTT are prolonged, and septic patients should be alerted to the possibility of severe AKI when RRI ≥ 0.695 or TTP ≥ 28.4 s [103]. Compared to non-septic shock patients, septic shock patients had a cortical PI parameter was low and MTT parameter was high, and higher in patients with severe AKI; MTT parameter may be a more accurate parameter to assess intrarenal hemodynamics. Similar conclusions have been drawn from comparisons in healthy volunteers versus patients with septic shock [10, 19]. A meta-analysis study demonstrated that reduced microcirculatory perfusion had occurred in AKI patients prior to the alteration of blood creatinine, as evidenced by prolonged perfusion time and reduced of renal cortical AS parameter [104]. TIC in non-AKI patients showed a slowing down after a rapid rise to peak, but AKI patients showed a slow rise to peak followed by a slow decline. The AKI 24-h group exhibited attenuated PI, prolonged PT, and decreased wash-in rate (WIR) compared to the non-AKI 24-h group. Significant differences were also found in day-7 [105]. Renal blood flow and time-averaged velocity were significantly decreased, PI was decreased, and TTP was prolonged between subgroups with exacerbation in the AKI group compared to the non-AKI group. Renal microcirculation PI and TTP parameters were independently and linearly correlated with blood creatinine [106]. The combination of blood creatinine, WIS and PI improved the accuracy of diagnosing septic AKI [107]. About the CEUS parameter relative blood volume (RBV) may be abnormal when renal perfusion reduction is more severe [108].

### Application of CEUS in assessing the effects of drugs on the kidney

CEUS has also been used in assessing the effects of drugs on renal microcirculatory blood flow. In patients with septic shock, the CEUS parameter PI was significantly higher in the Terlipressin group than in the control group at 24 h after enrolment. Terlipressin improves renal perfusion in patients with septic shock [109]. Angiotensin II (Ang II) decreases renal cortical PI parameter in humans and the decrease in PI is more pronounced at higher doses, while the opposite is true for Captopril [110]. PI parameter was significantly reduced in healthy populations taking diclofenac sodium [111]. In assessing the role of curcumin in cecum ligation and puncture (CLP) rats, it was found that CEUS parameters PI, DT/2, and AUC were elevated in the curcumin-treated group compared to the CLP group [112]. Dexmedetomidine was found to reduce the incidence of postoperative AKI in non-cardiac postoperative patients [113]. Animal studies have shown that dexmedetomidine significantly improves renal microcirculation in I/R rabbits [114]. Assessment of renal cortical perfusion during I/R in rabbits revealed that AT and TTP values peaked 3 d after I/R surgery and correlated with the most significant pathological changes at the same timepoint [69]. In the I/R cat model, infusion of Ang II resulted in enhanced mean peak renal values, significant decreases in wash-in perfusion index (WiPI) and wash-out rate (WOR), and a trend towards lower wash-in area under the curve (WiAUC) [115]. A porcine model of severe hemodilution found that the use of hydroxyl ethyl starch preserved intrarenal microcirculatory perfusion and renal function [116]. In healthy rabbits, the CEUS parameters TTP and AUC increased significantly while arrival time (AT) and descending time (DT) decreased slightly within 6 h after intramuscular injection of nitroglycerin [117]. The use of hydrocortisone in a dog model resulted in a significant increase in PI parameter in renal cortical and medullary [118].

### Clinical application of CEUS and its advantages

CEUS predicts both the occurrence and prognosis of AKI. CEUS assessment suggested that patients at risk of AKI had reduced renal perfusion within 24 h of surgery. Compared with baseline, there was no overall difference in median PI on ICU admission. However, the day after surgery, median PI had decreased by 50%; 48% increase in MTT, both suggestive of decreased perfusion. These differences persisted after correction for hemoglobin; vasopressors use and mean arterial pressure [119]. This suggests to us that monitoring CEUS within 24 h of ICU admission is useful in the clinic. Studies that have also performed continuous CEUS renal monitoring have found that for macro-circulation and other microcirculation indices, renal microcirculation impairment lasted longest (Day 4 was still relevant) [19]. It is meaningful to monitor renal microcirculatory blood flow once a day for 72 h, and comparisons of CEUS parameters still differed between the 120 h groups. Whether assessing renal microcirculatory blood flow with CEUS at times beyond 4 days is meaningful remains to be investigated. Renal microcirculation monitoring in hemodynamically unstable patients still requires individualized monitoring and real-time adjustment of the number and frequency of monitor.

Previous studies have suggested that renal perfusion pressure is determined by MAP–CVP [120]. However, recent study had found that CEUS suggested that renal microcirculatory blood flow was different from macro-circulatory blood flow and other microcirculatory blood flow [19]. CEUS provides a clear picture of blood flow in the renal microcirculation, allows real time, multiple, and as-needed monitoring of the renal microcirculation at the bedside with no renal function side effects, besides, it can distinguish between cortical and medullary blood flow in the kidney. Of course, CEUS still has its problems, the lack of large-scale clinical studies to give international standards, operator differences, patient differences, etc., still need more and more large-scale clinical studies to further confirm.

Renal cortical and medullary blood flow varies in different diseases and CEUS can assist in assessing renal regional blood flow. Renal regional blood flow studies using CEUS in ICU patients are still rare and highly variable. The influence of the arterial and venous ends on the microcirculation and the intricate relationship between them remain unclear. Monitoring intrarenal blood flow, combined with the characteristics of the macro-circulatory hemodynamics is important in the finding the hemodynamic etiology of AKI patients is crucial.

In summary, the assessment and treatment of renal blood flow in patients with severe disease-related AKI is of priority and necessity, and the alteration of renal hemodynamics with microcirculation as the core is the key to the occurrence, development and prognosis of severe disease-related AKI; while microcirculation perfusion in different parts of the kidney is different and should be assessed separately; the comprehensive assessment of renal macro-circulation and microcirculation is important for the clinical adjustment of renal hemodynamic treatment in patients with AKI. CEUS can assess the microcirculation perfusion in different parts of the kidney, is non-invasive, implementable, safe and convenient, and realizes the visualization of bedside renal perfusion imaging, which provides a new method for identifying the high-risk group of AKI as early as possible and avoiding the occurrence and development of AKI, and it is an important means for ICU patients to realize the individualized and organized treatment. At present, CEUS still has its shortcomings, the safety of its application in critically ill patients needs to be further evaluated, and the quantitative analysis is affected by many factors, for which there is no reliable method to overcome, such as tissue harmonic interference, affected by respiratory motion, body mass index, depth, etc., which can only be quantitatively studied in a certain cross section, the dosage and mode of administration of contrast medium, the ultrasound parameter settings, and the significance of the parameters for quantitative analysis. In addition, there is a lack of international standards for quantitative assessment of renal microcirculation with ultrasonography-related data, and large international clinical trials are needed to determine the optimal parameters for clinical assessment and the normal ranges of relevant parameters in different subjects. Therefore, the use of renal ultrasonography for the assessment of renal microcirculation requires strict procedures and careful interpretation of the meaning of the data. More data from CEUS clinical evaluations could provide clinical data to establish appropriate standards.

## Data Availability

The data set used and analyzed for the current study is available from the corresponding author on reasonable request.

## Abbreviations

AKI: Acute kidney injury
Ang II: Angiotensin II
AS: Ascending slope
AT: Arrival time
a.u.: Arbitrary units
AUC: Area under the curve
CEUS: Contrast-enhanced ultrasound
CLP: Cecum ligation and puncture
CO: Cardiac output
CVP: Central venous pressure
DS: Descending slope
DT/2: Descending time/2
FT: Fall time
GFR: Glomerular filtration rate
IAP: Abdominal pressures
ICU: Intensive care unit
IRP: Renal interstitial pressure
MAP: Mean arterial pressure
MI: Mechanical index
MTT: Mean transit time
PE: Peak enhancement
PI: Peak intensity
PIT: Peak intensity time
PT: Peak time
PO_2_: Pressure of oxygen
PuO_2_: Pressure of urine oxygen
RBF: Renal blood flow
RBV: Relative blood volume
ROI: Region of interest
ROS: Reactive oxygen species
RRI: Renal arteriolar resistance
RRT: Renal replacement therapy
RT: Rise time
RVP: Renal venous pressure
TIC: Time–intensity curve
TTP: Time to peak
UCA: Ultrasound contrast agent
WiAUC: Wash-in area under the curve
WiPI: Wash-in perfusion index
WIR: Wash-in rate
WIS: Wash-in slope
WIT: Wash-in time
WiWoAUC: Wash-in and wash-out area under the curve
WoAUC: Wash-out area under the curve
WOR: Wash-out rate
WOT: Wash-out time
